# Supplementary material for: Perilipin 5 links mitochondrial uncoupled respiration in brown fat to healthy white fat remodeling and systemic glucose tolerance
Source: Nat Commun. 2021 Jun 3;12:3320. doi: 10.1038/s41467-021-23601-2 (PMC8175597; doi:10.1038/s41467-021-23601-2)
Supplement: Supplementary file 1 — Supplementary Information file [file 41467_2021_23601_MOESM1_ESM.pdf]

Supplementary Information File

Supplementary Figure 1

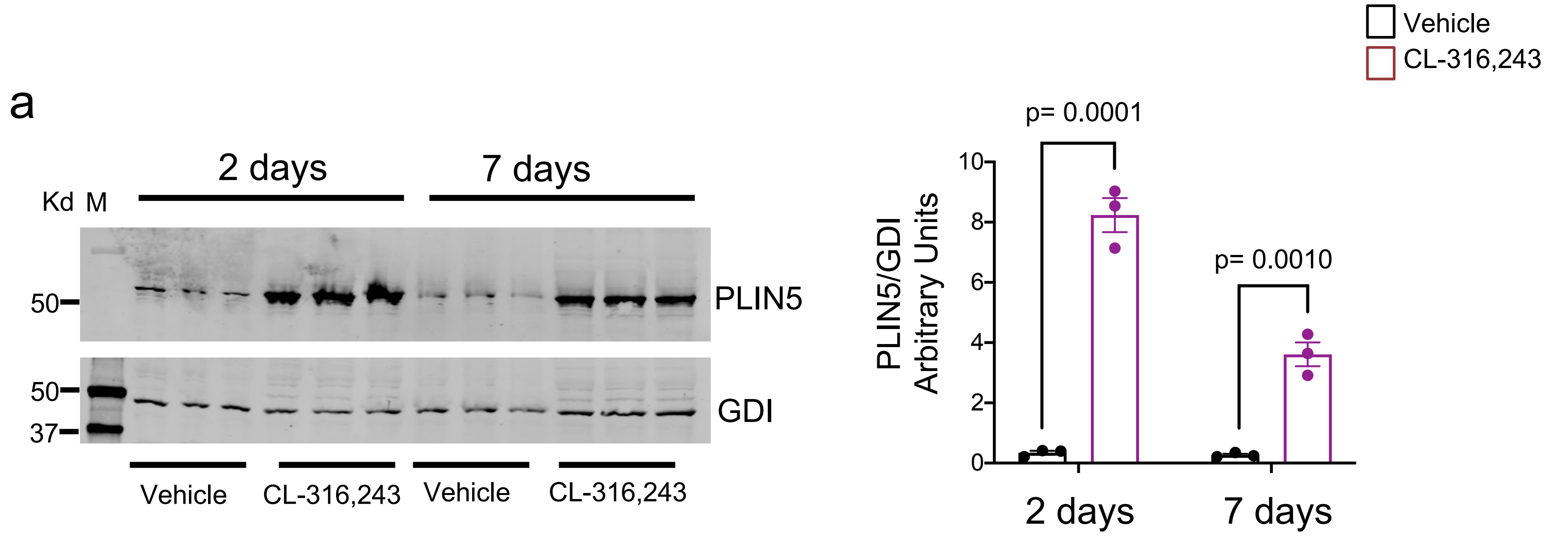

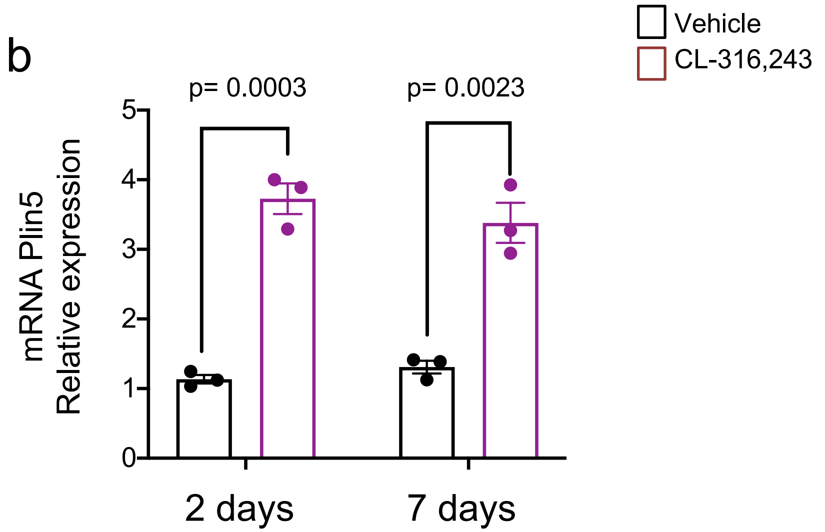

Supplementary Figure 1. BAT PLIN5 expression increases with systemic administration of CL-216,243

a. WB for PLIN5 in BAT from wild type mice (C56BL/6J) treated with CL-216, 243 or Vehicle for 2 or 7 days (left panel) WB quantification (right panel). *n*=3 mice per group

b. qPCR for *Plin5* relative mRNA expression from wild type mice (C56BL/6J) treated with CL-216, 243 or Vehicle for 2 or 7 days. *n*=3 mice per group

For panels a and b values are mean ± s.e.m and statistical analysis was performed using unpaired two tailed Student t test adjusted for multiple comparisons using Holm Sidák method. Source data are provided as a Source Data file.

Supplementary Figure 2

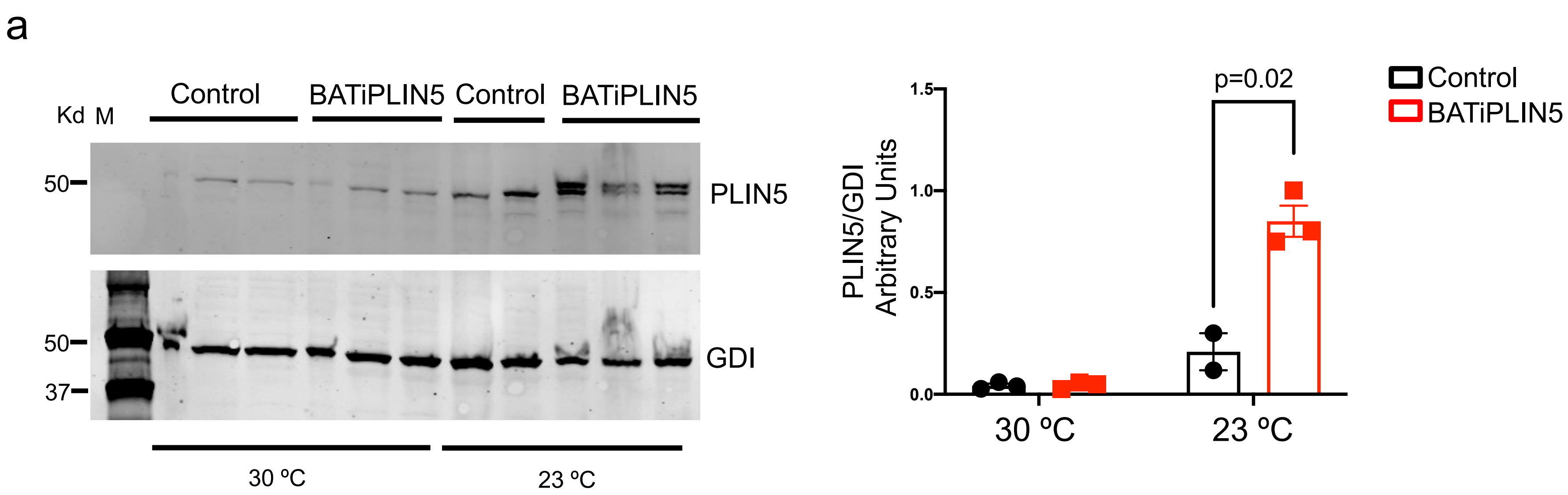

Supplementary Figure 2. PLIN5 protein expression is not increased in the BATiPLIN5 mice at thermoneutrality

a. WB for PLIN5 in BAT from Control or BATiPLIN5 mice housed at the indicated temperatures for 7 days. *n*=3 mice per group

Values are mean ± s.e.m and statistical analysis was performed using unpaired two tailed Student t test adjusted for multiple comparisons using Holm Sidák method. Source data are provided as a Source Data file.

Supplementary Figure 3

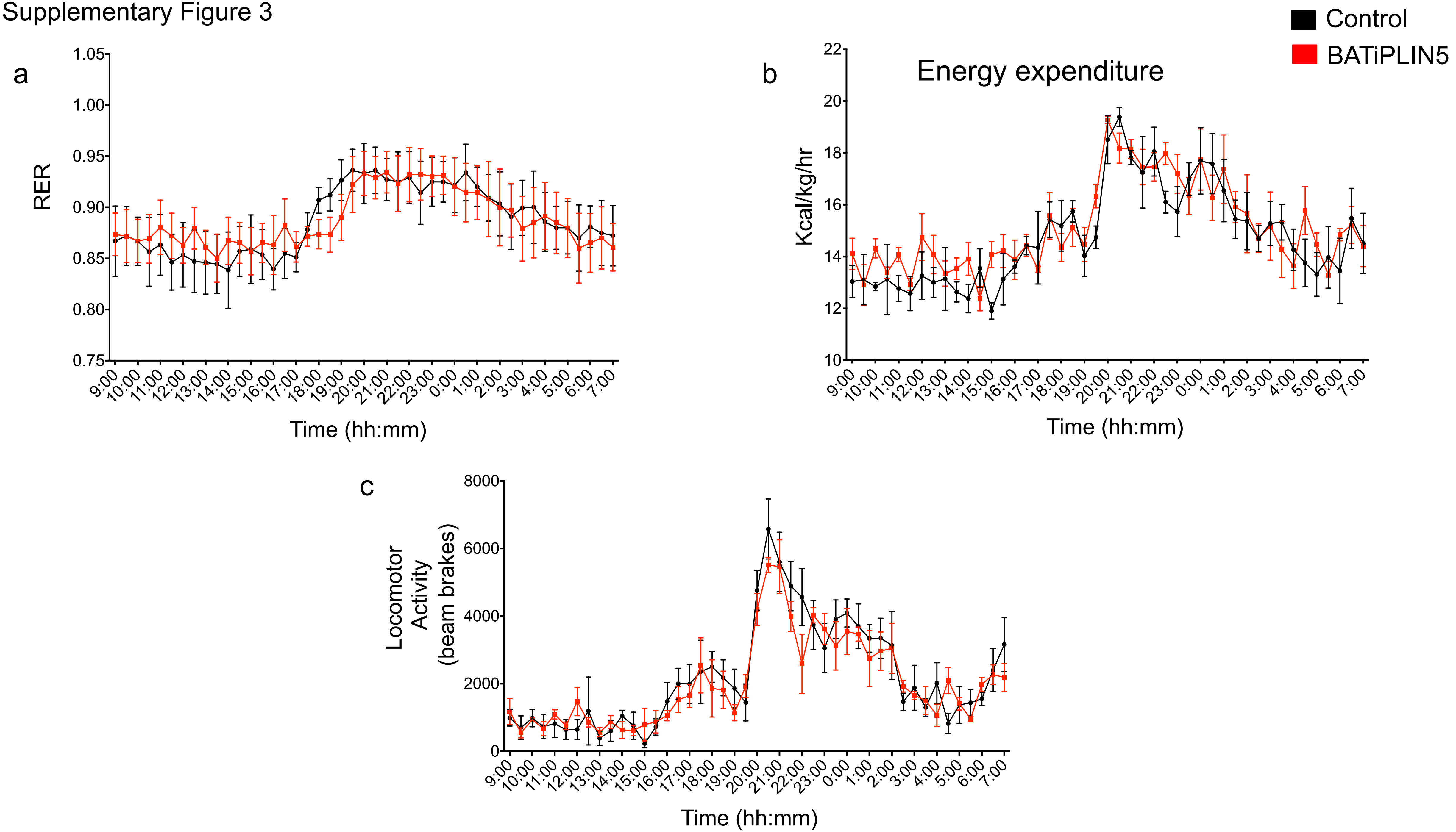

Supplementary Figure 3. Respiratory exchange ratio, energy expenditure and locomotor activity in BATiPLIN5 and Control mice

a. Respiratory exchange ratio (RER) from BATiPLIN5 or Control mice housed at 23 ºC. *n*=5 mice per group

b. Energy expenditure from BATiPLIN5 or Control mice housed at 23 ºC. *n*=5 mice per group

c. Locomotor activity (beams breaks) from BATiPLIN5 or Control mice housed at 23 ºC. *n*=5 mice per group

Values are mean ± s.e.m. Source data are provided as a Source Data file.

Supplementary Figure 4

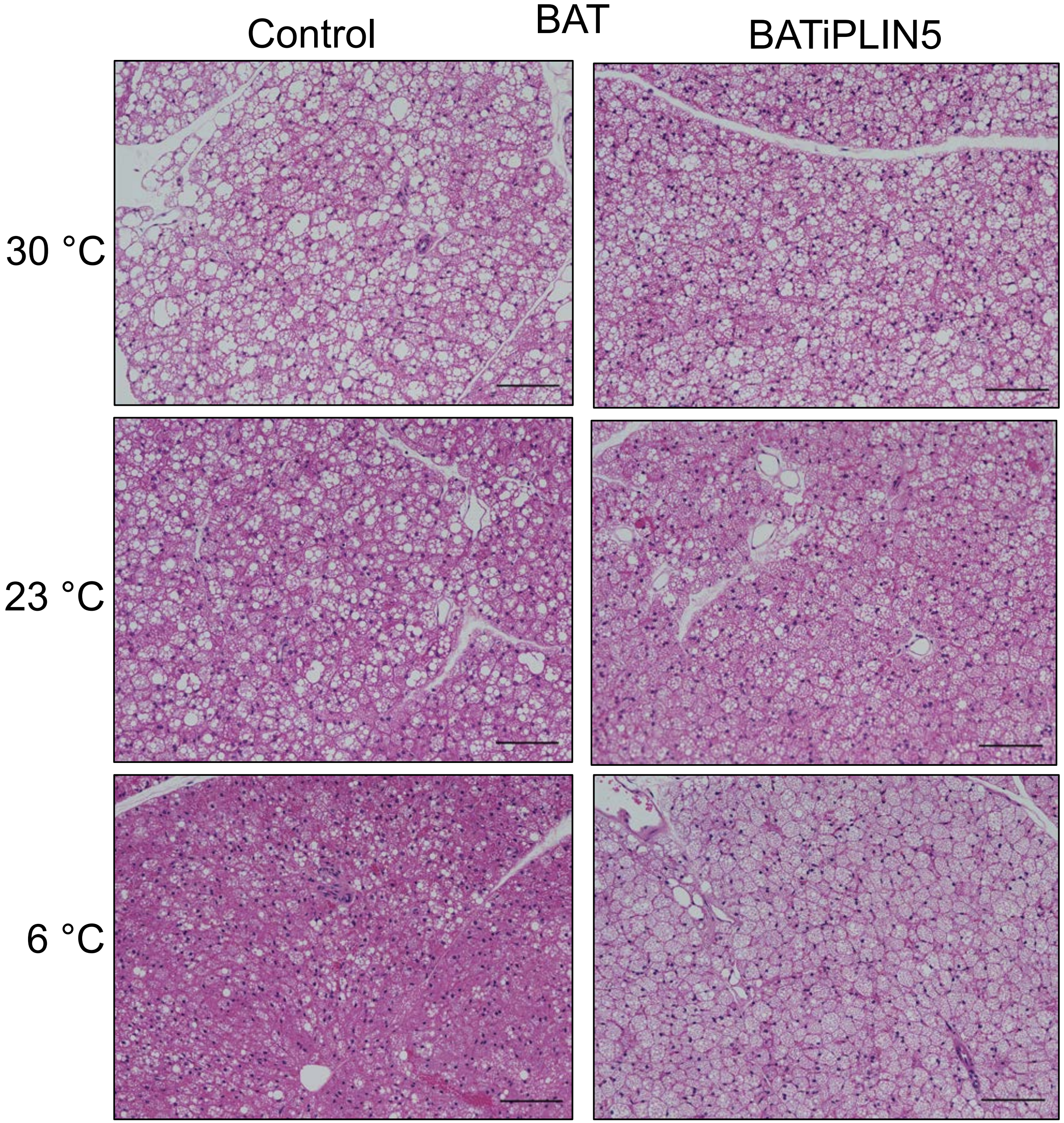

Supplementary Figure 4. Hematoxylin and eosin staining of BAT from BATiPLIN5 and Control mice

a. Representative images of hematoxylin and eosin staining of BAT from Control or BATiPLIN5 mice housed at 23 ºC or exposed overnight to 30 ºC or 6 ºC. Scale bar=100 µm.

Supplementary Figure 5

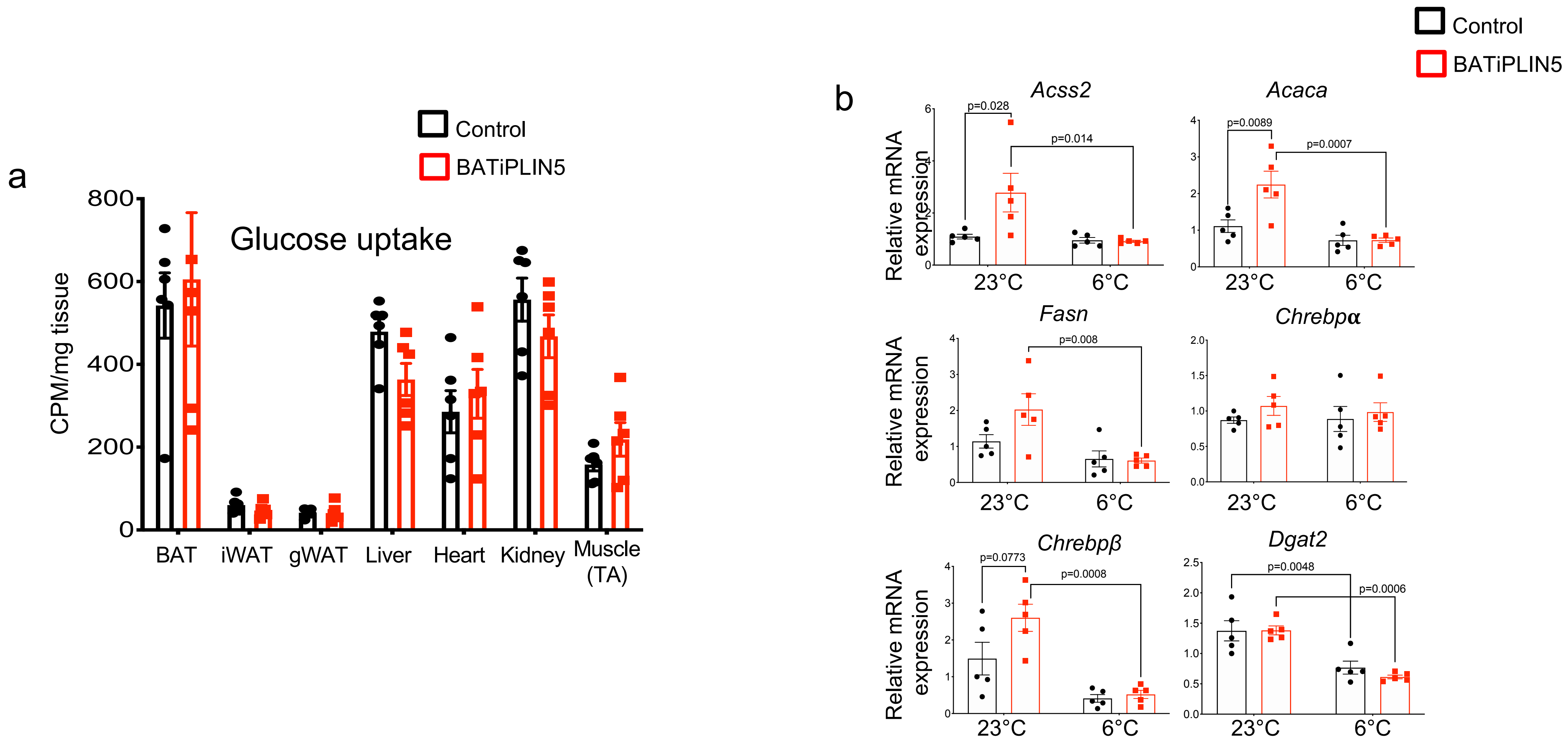

Supplementary Figure 5. Tissue glucose uptake and lipogenesis gene expression in BAT from BATiPLIN5 and Control mice

a. Deoxy-D-glucose,2-[1-14] uptake after oral gavage in the indicated tissues. Values are mean ± s.e.m *n*=6 mice per group.

b. qPCR for the indicated genes relative mRNA expression in BAT from Control or BATiPLIN5 mice housed at 23 ºC or exposed 6 ºC for 16 hours. Values are mean ± s.e.m. *n*=3 mice per group. Statistical analysis was performed using two-way ANOVA followed by Tukey post-test. P values are shown in the Figure. Source data are provided as a Source Data file.

Supplementary Figure 6

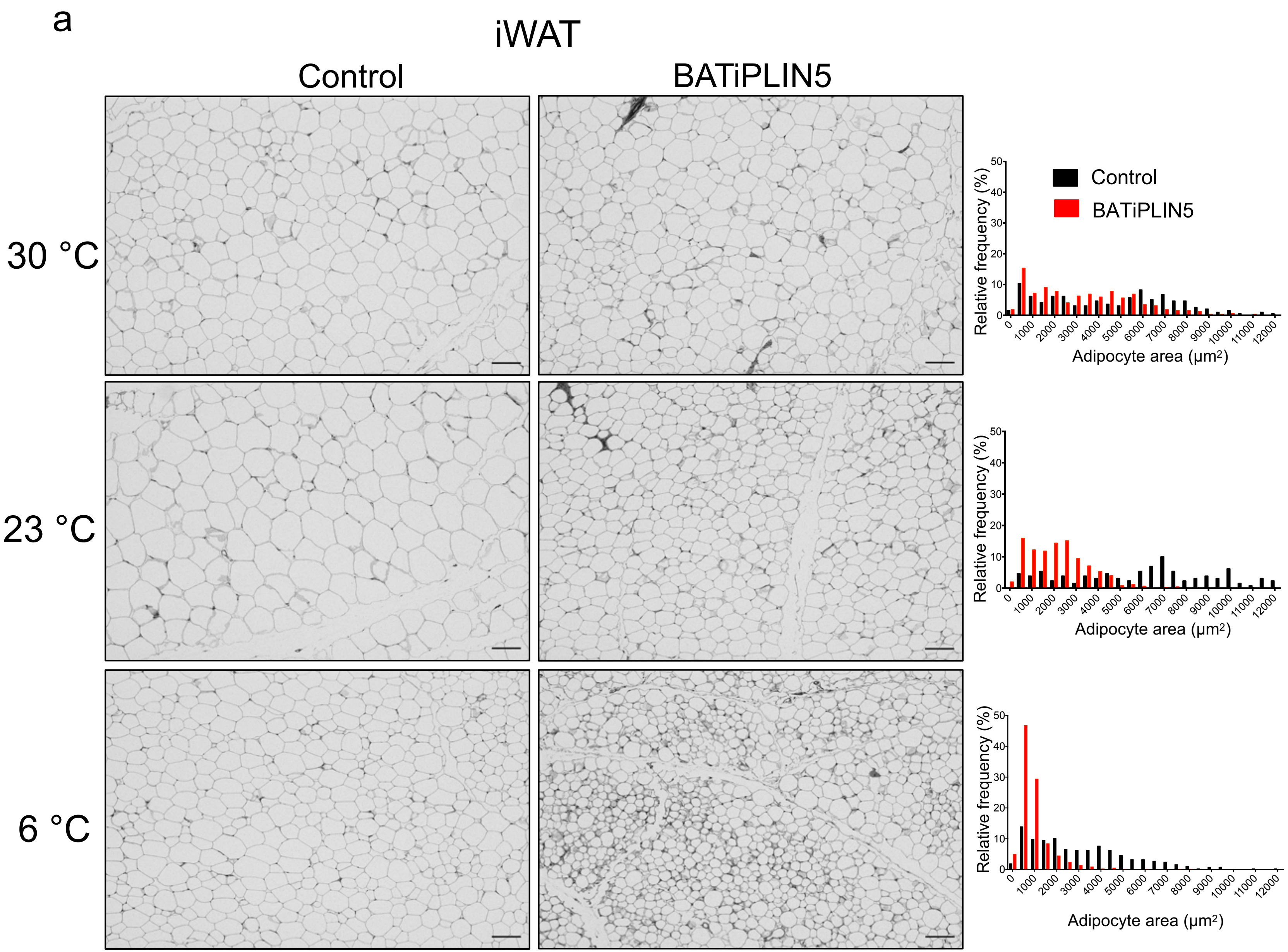

Supplementary Figure 6. Hematoxylin and eosin staining of iWAT from BATiPLIN5 and Control mice with adipocyte size quantification

a. Representative images of hematoxylin and eosin staining of white adipose tissue from Control or BATiPLIN5 mice housed at 23 ºC or exposed overnight to 30 ºC or 6 ºC and adipocyte size quantification expressed as relative frequency . Scale bar=100 µm. Source data are provided as a Source Data file.

Supplementary Figure 7

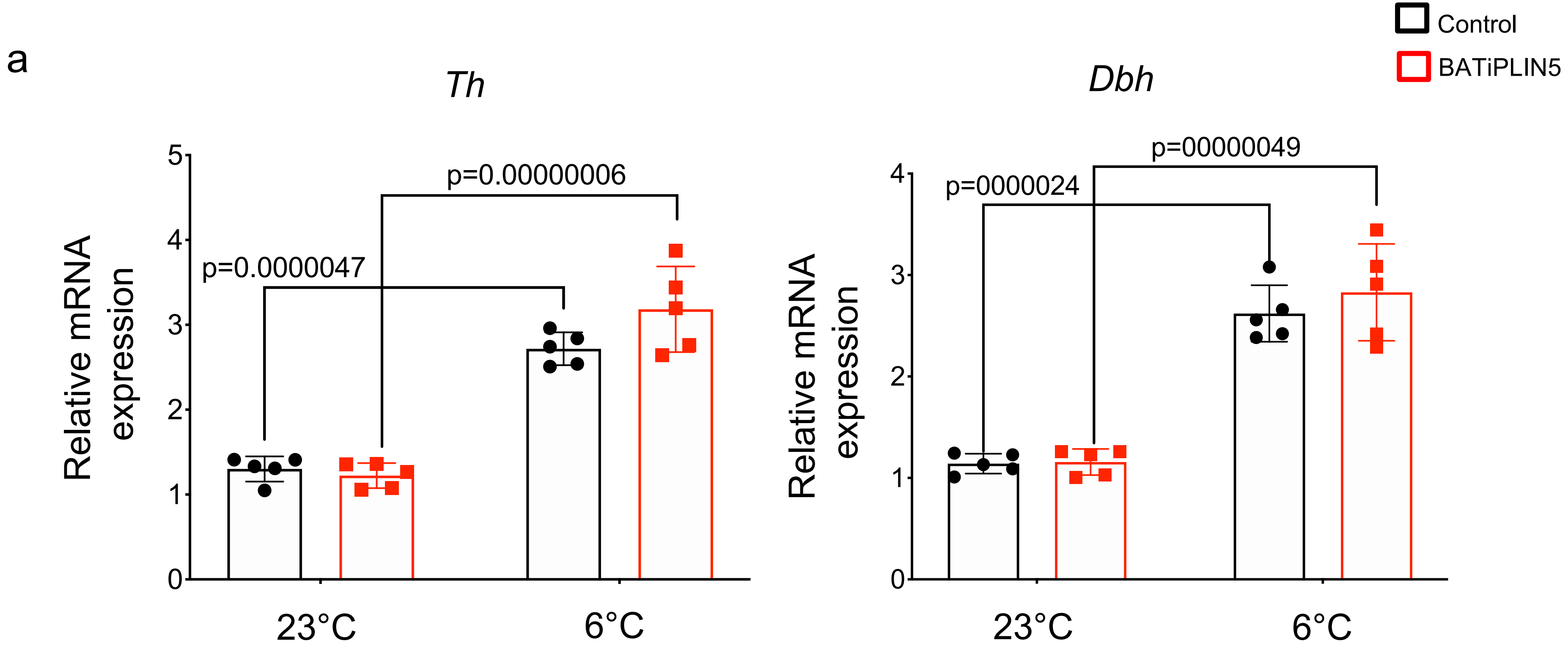

Supplementary Figure 7. Tyrosine 3-hydroxylase (*Th*) and Dopamine beta-hydroxylase (*Dbh*) gene expression in iWAT of BATiPLIN5 and Control mice

a. qPCR for the indicated genes relative mRNA expression in iWAT from Control or BATiPLIN5 mice housed at 23 ºC or exposed 6 ºC for 16 hours. Values are mean ± s.e.m. *n*=3 mice per group. Statistical analysis was performed using two-way ANOVA followed by Tukey post-test. P values are shown in the Figure. Source data are provided as a Source Data file.

Supplementary Figure 8

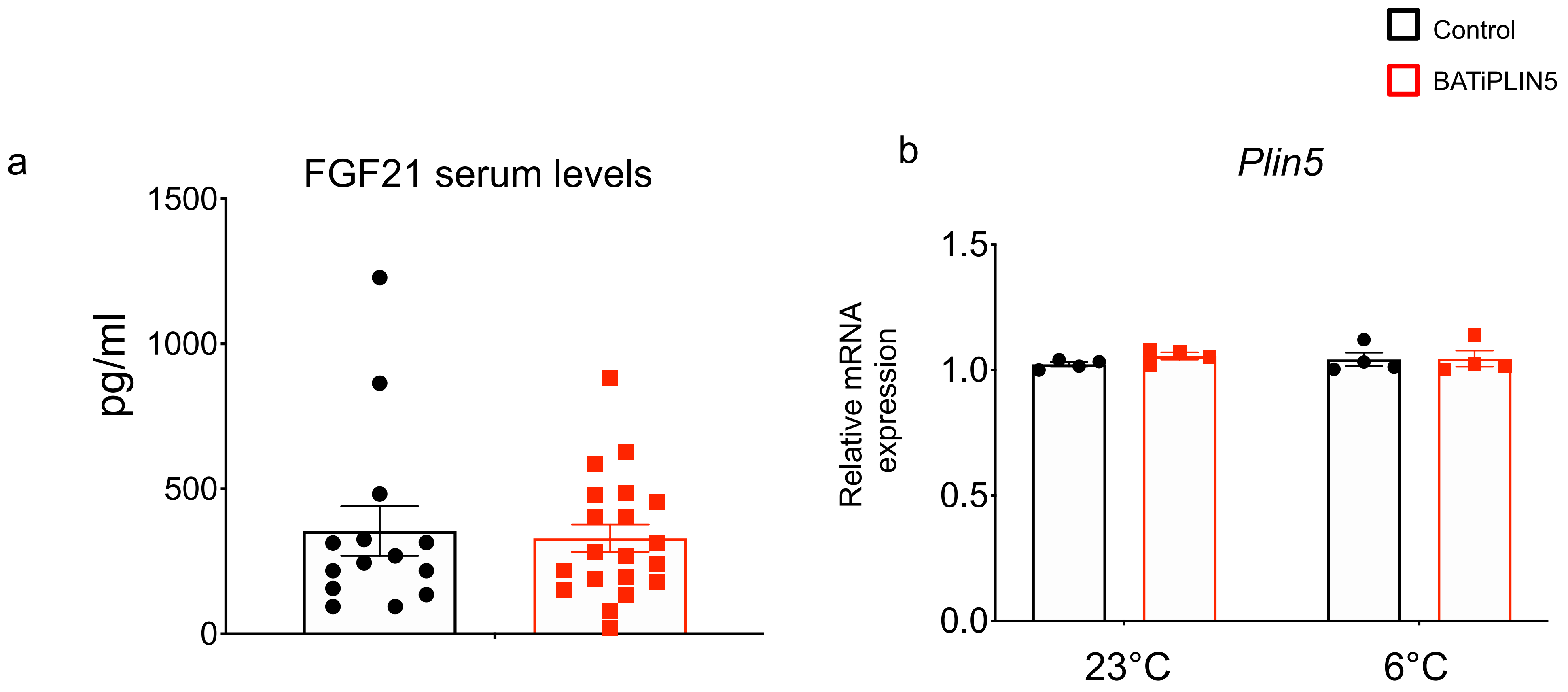

Supplementary Figure 8. FGF21 serum levels and gWAT *Plin5* gene expression from BATiPLIN5 and Control mice

a. Fasting Fgf21 serum levels in Control or BATiPLIN5 mice housed at 23º C (Values are mean ± s.e.m *n*= 14 mice for Control group and 20 mice for BATiPLIN5 group)

b. qPCR for *Plin5* relative mRNA expression in gWAT from Control or BATiPLIN5 mice housed at 23 ºC or exposed 6 ºC for 16 hours. Values are mean ± s.e.m. *n*=4 mice per group.

Source data are provided as a Source Data file.

Supplementary Figure 9

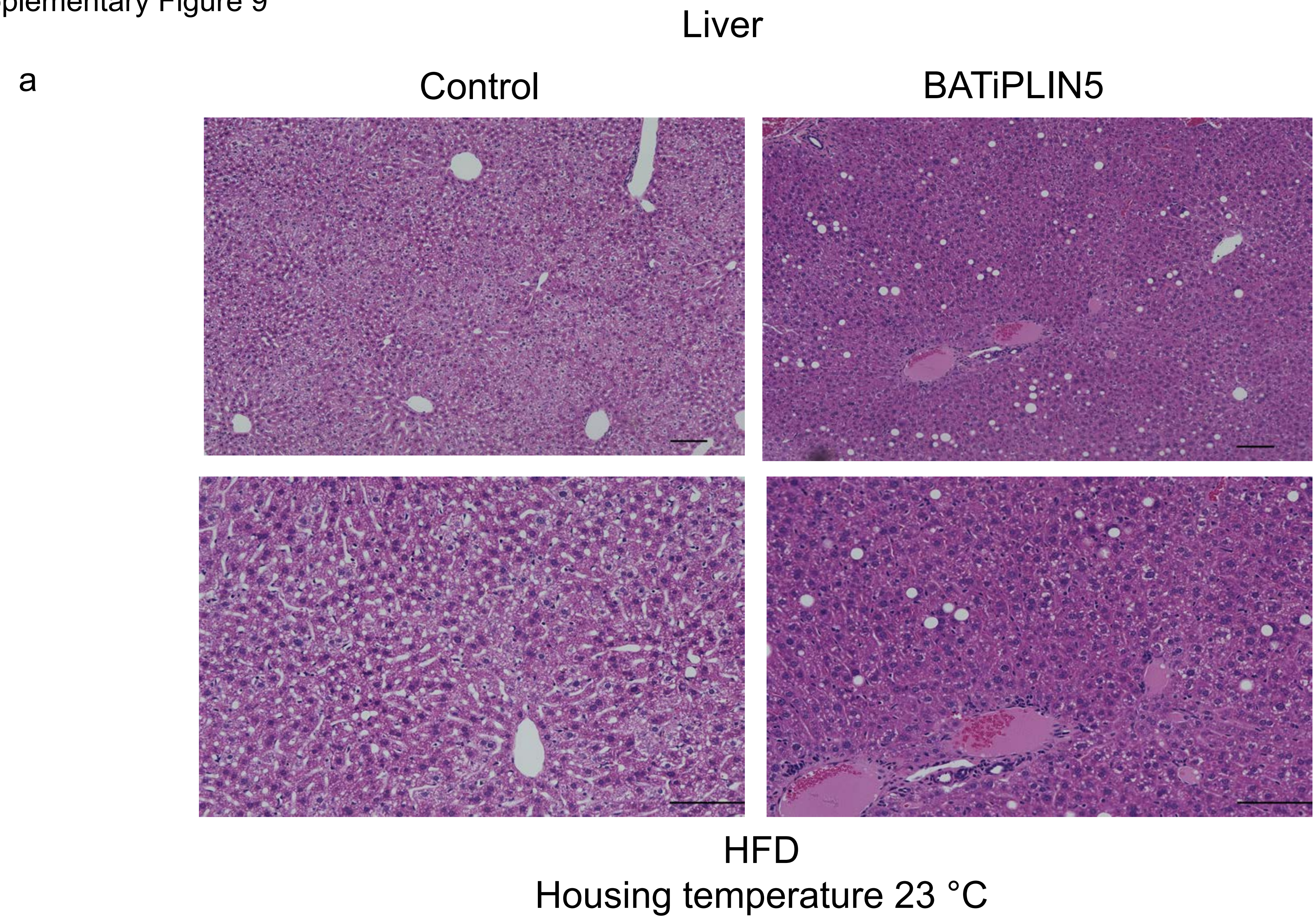

Supplementary Figure 9. Hematoxylin and eosin staining of liver from BATiPLIN5 and Control mice

a. Representative images of hematoxylin and eosin staining from fasted Control or BATiPLIN5 mice fed with HF diet for 12 weeks. Scale bar=100 μm.

Supplementary Figure 10

a

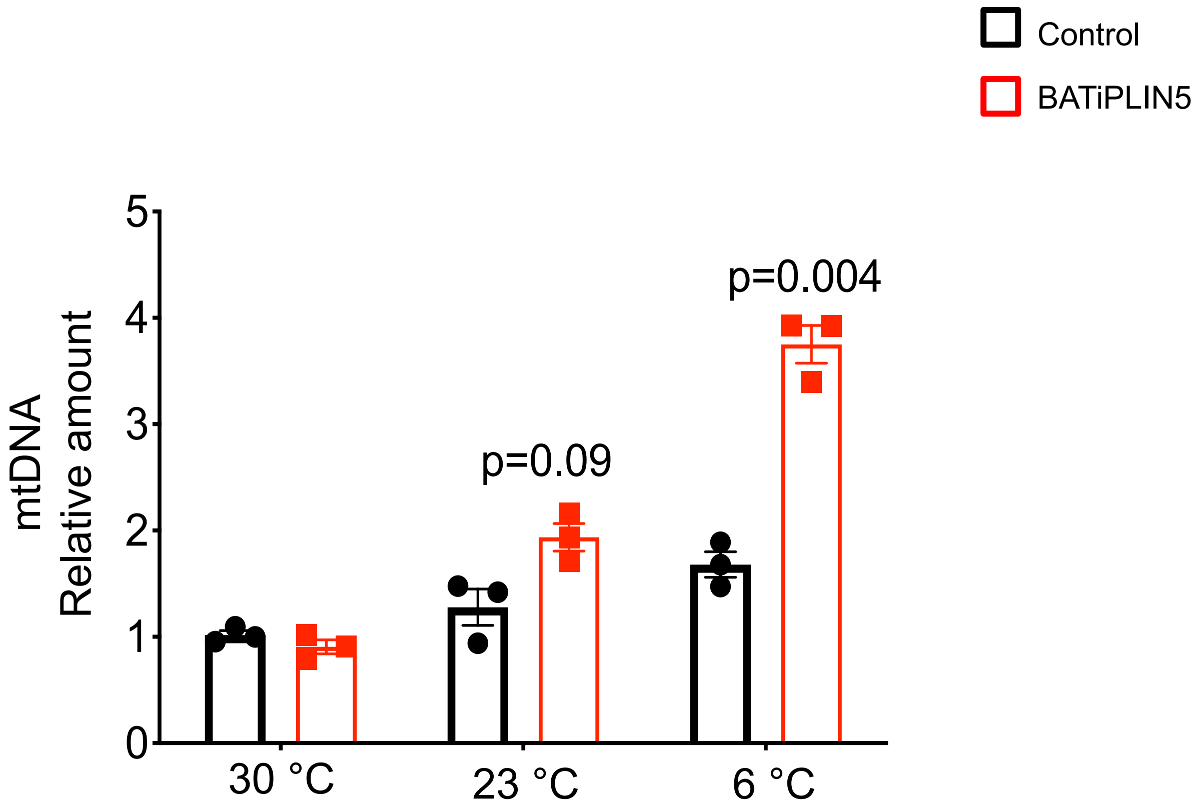

Supplementary Figure 10. Mitochondrial DNA quantification of BAT from BATiPLIN5 and Control mice

a. Relative amount of BAT mitochondrial DNA from Control or BATiPLIN5 mice housed at 23 ºC or exposed to the indicated temperatures for 16 hours. *n*=3 mice per group.

Values are mean ± s.e.m and statistical analysis was performed using two tailed Student t test adjusted for multiple comparisons using Holm Sidák method. Source data are provided as a Source Data file.

Supplementary Figure 11

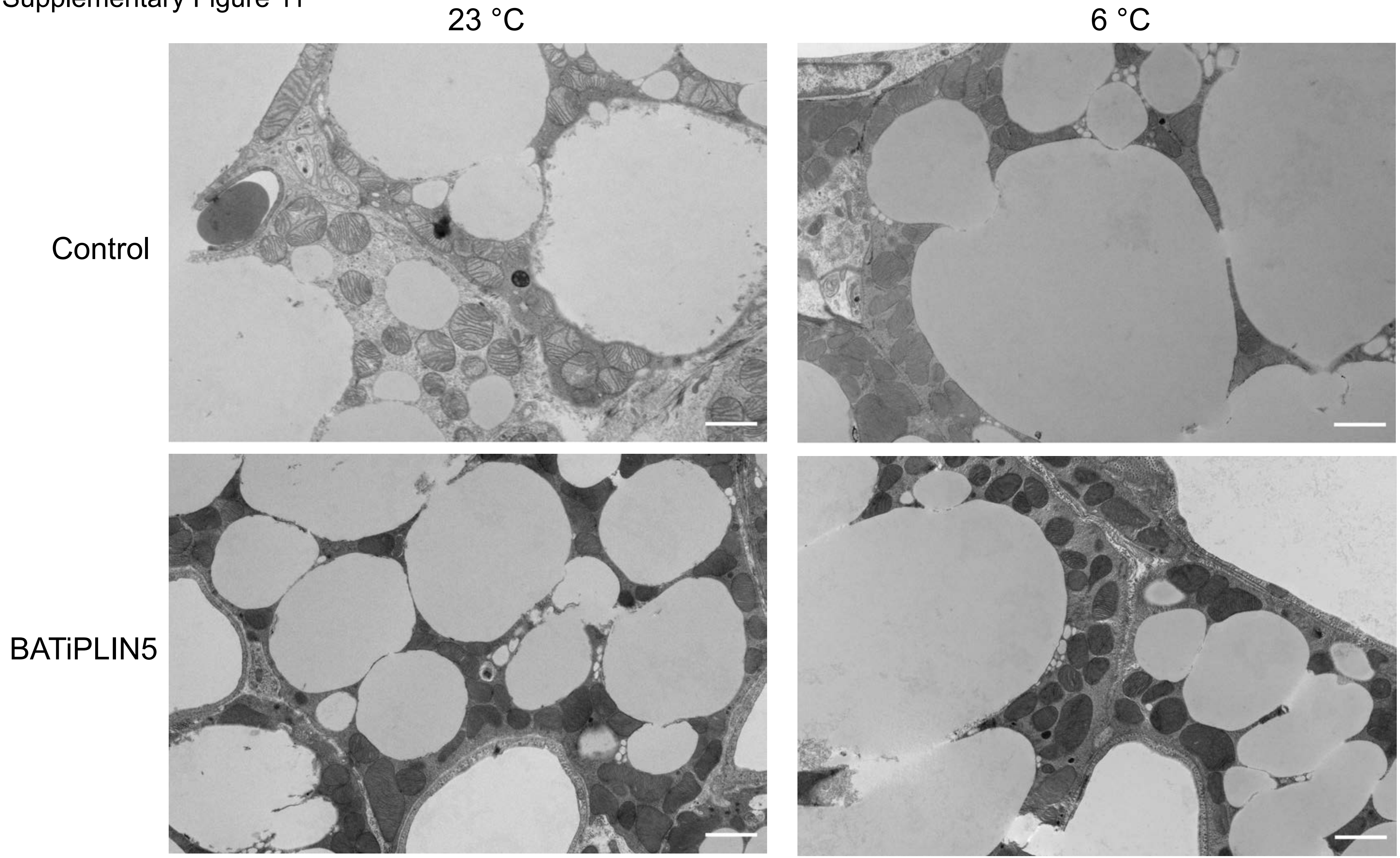

Supplementary Figure 11. Electron microscopy (EM) of BAT from BATiPLIN5 and Control mice

a. Additional representative images of BAT electron microscopy (EM) from Control or BATiPLIN5 mice housed at 23 ºC or exposed to 6 ºC for 16 hours. Scale bar= 2 μm.

Supplementary Figure 12

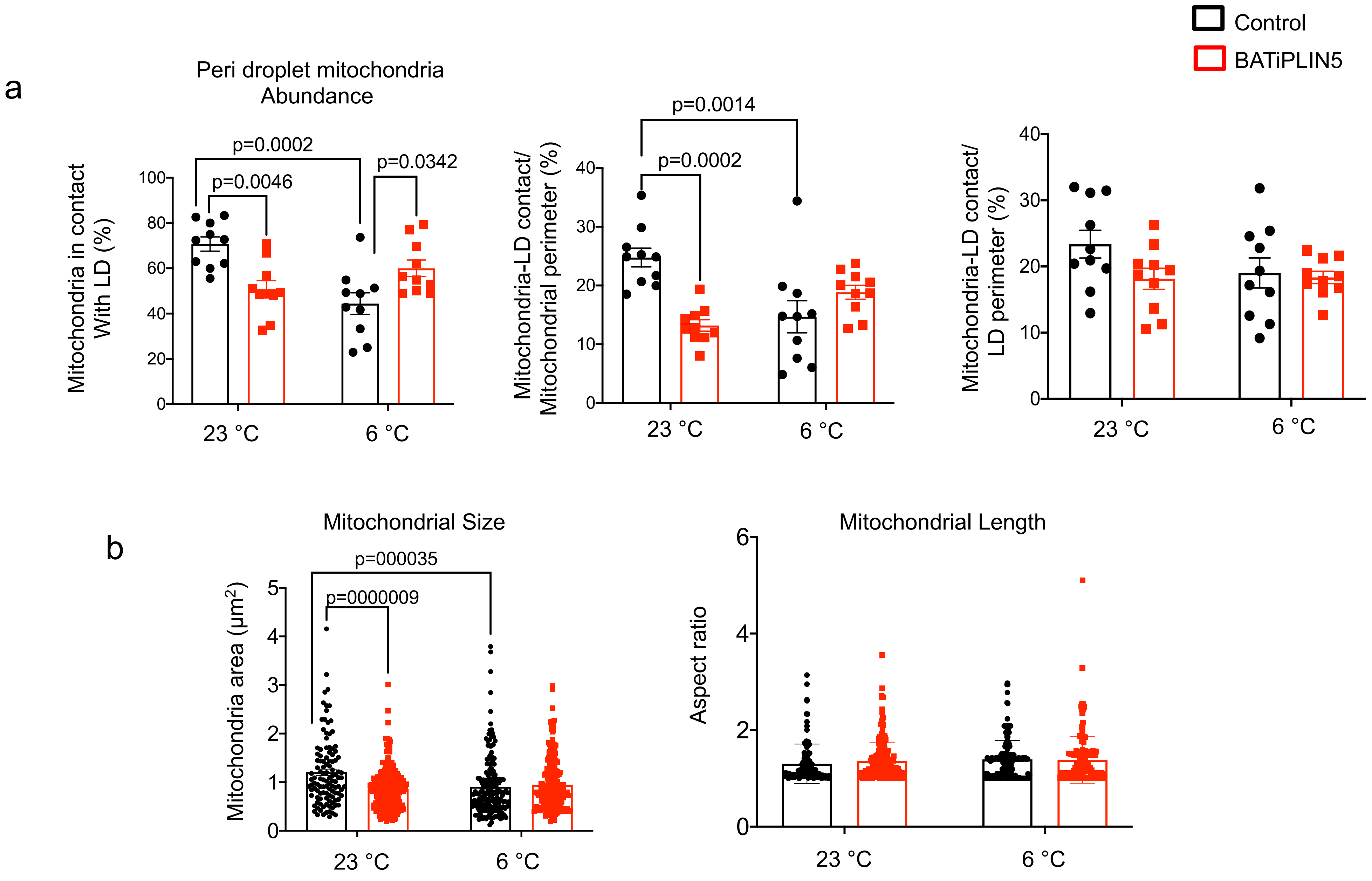

Supplementary Figure 12. LD-mitochondria contacts and mitochondria size quantification of BAT from BATiPLIN5 and Control mice

a. Mitochondria in contact with lipid droplets quantified by count (left), % of mitochondrial perimeter (center) and % of lipid droplet perimeter (right) *n*=10 EM fields per condition.

b. Mitochondrial area (left panel) and mitochondrial length (right panel). *n*=129-212 mitochondria-all the mitochondria in 5 EM.

Values are mean ± s.e.m. For panels a and b statistical analysis was performed using two-way ANOVA followed by Tukey post-test. P values are shown in the figures. Source data are provided as a Source Data file.

Supplementary Figure 13

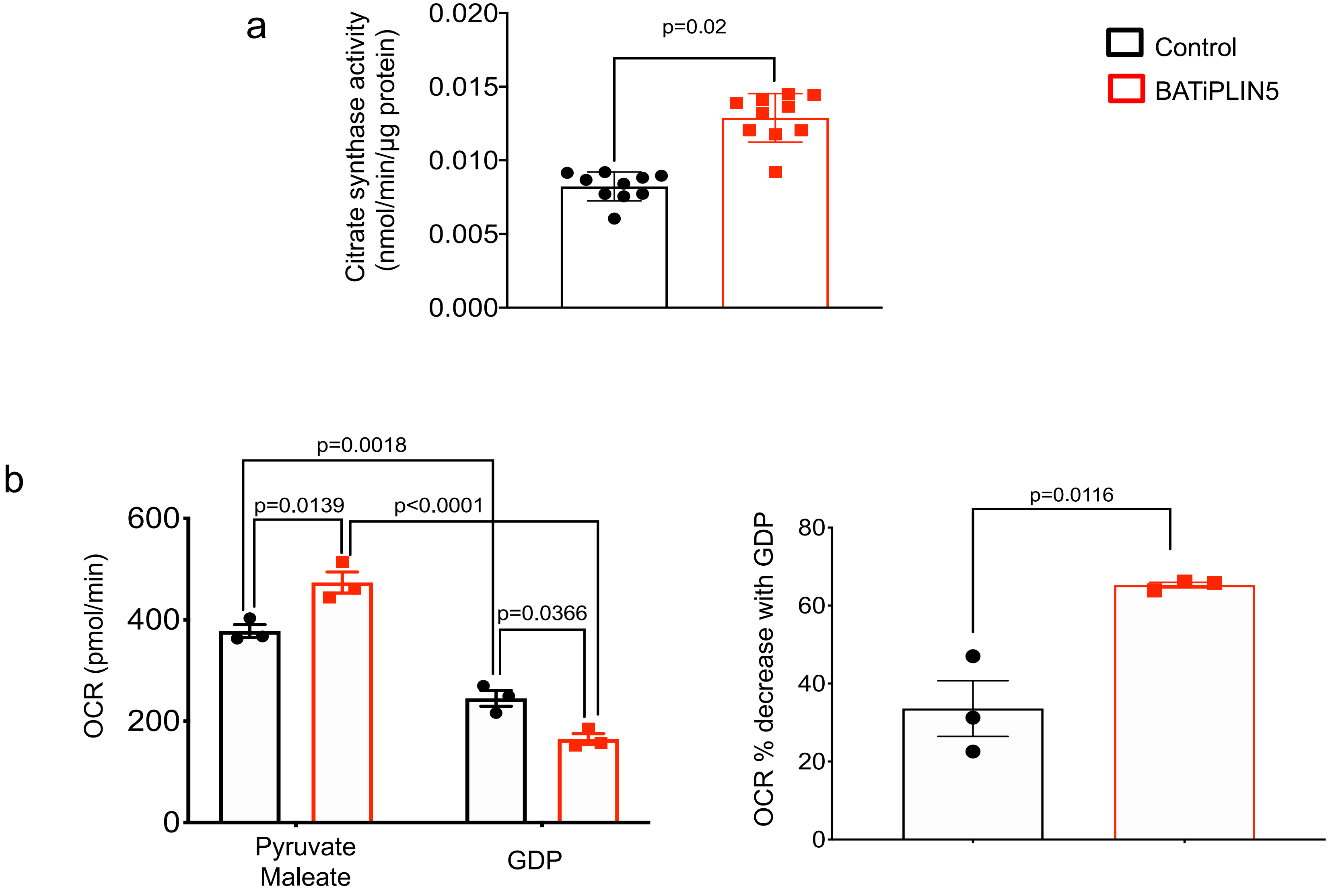

Supplementary Figure 13. Citrate synthase activity and OCR measurements of BAT from BATiPLIN5 and Control mice

a. Citrate synthase activity from BAT cell lysates obtained from Control and BATiPLIN5 mice housed at 23 ºC. *n*=10 mice per group.

b. Average of the 3 measurements after pyruvate/maleate injection and GDP injection (left panel). Percentage of decrease on OCR after GDP injection (right panel). *n*=3 mice per group.

Values are mean ± s.e.m. For panel a and b (right) statistical analysis was performed using unpaired two-sided Student t-test. P values are shown in the figures. For panel b (left) statistical analysis was performed using two-way ANOVA followed by Tukey post-test. Source data are provided as a Source Data file.

Supplementary Figure 14

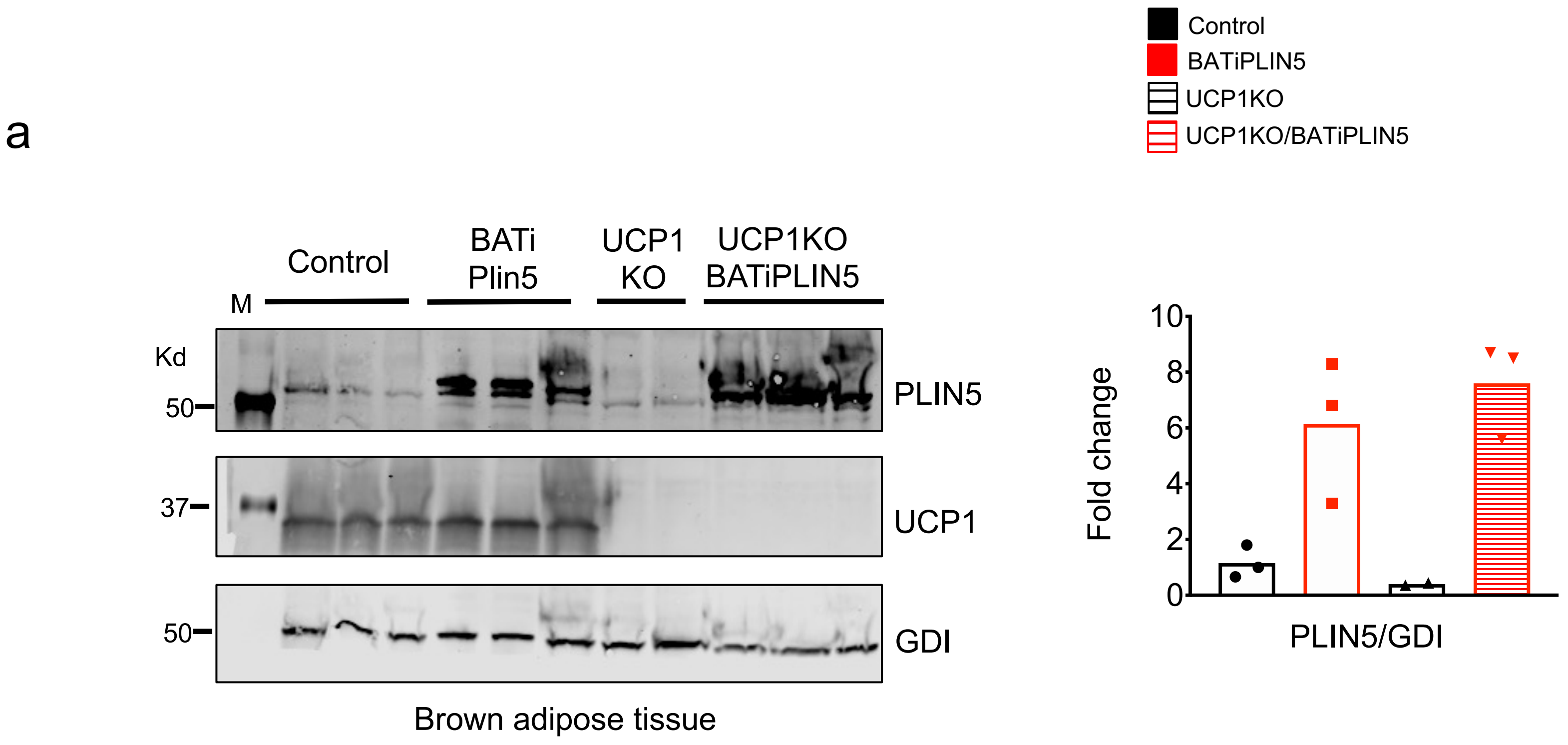

Supplementary Figure 14. PLIN5 and UCP1 protein expression from UCP1KO-BATiPLIN5 mice line

a. BAT WB for the indicated proteins from Control, BATiPLIN5, UCP1KO and UCP1KO-BATiPLIN5 mice (left panel) and quantification (right panel). *n*=3 mice for Control, BATiPLIN5 and UCP1KO-BATiPLIN5 and *n*=2 mice for UCP1KO

Source data are provided as a Source Data file.

Supplementary Figure 15

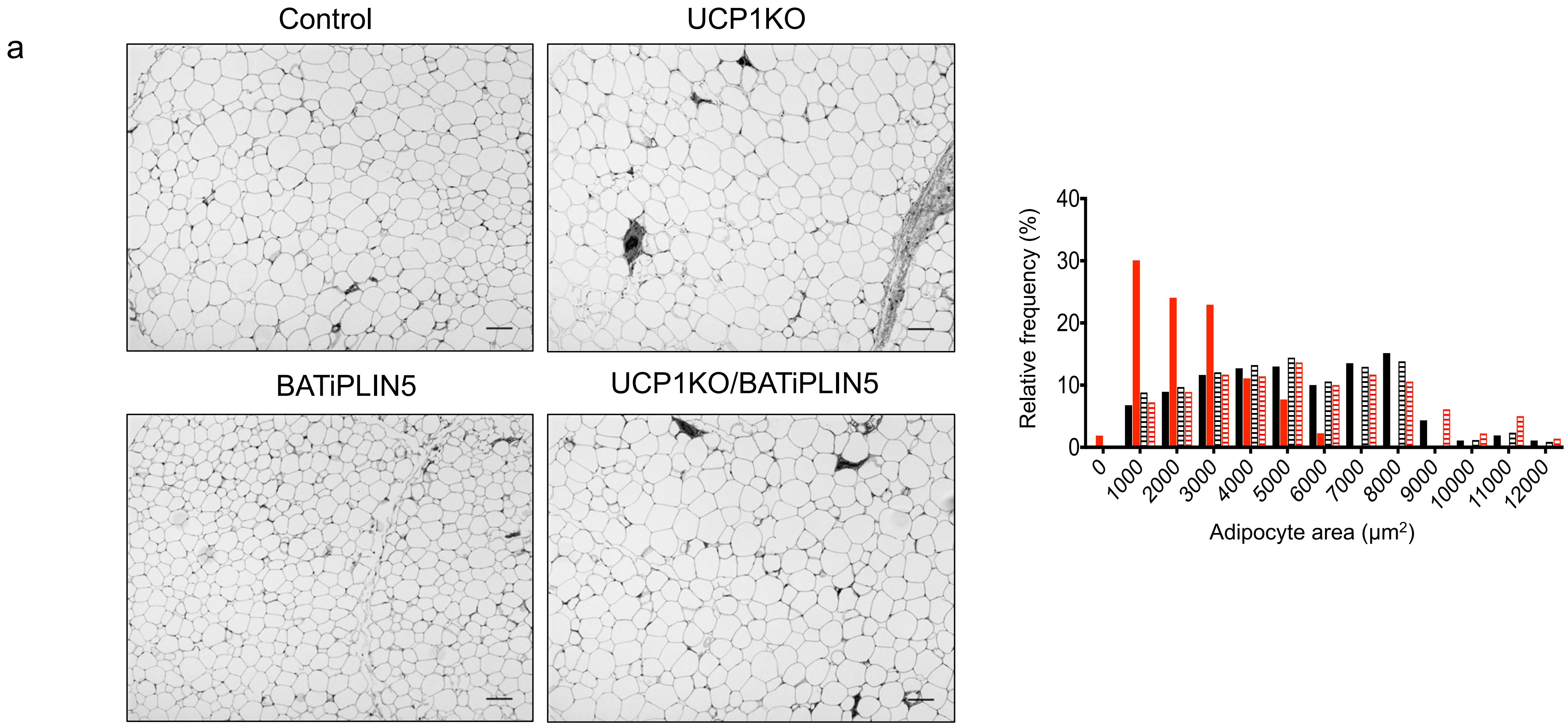

Supplementary Figure 15. Hematoxylin and eosin staining of iWAT from UCP1KO-BATiPLIN5 mice line

a. Representative image of hematoxylin and eosin staining of iWAT from Control, BATiPLIN5, UCP1KO and UCP1KO/BATiPLIN5 mice housed at 23 ºC (left panel) and adipocyte area percentage of frequency distribution (right panel). Scale bar= 50 mm

Source data are provided as a Source Data file.

Supplementary Figure 16

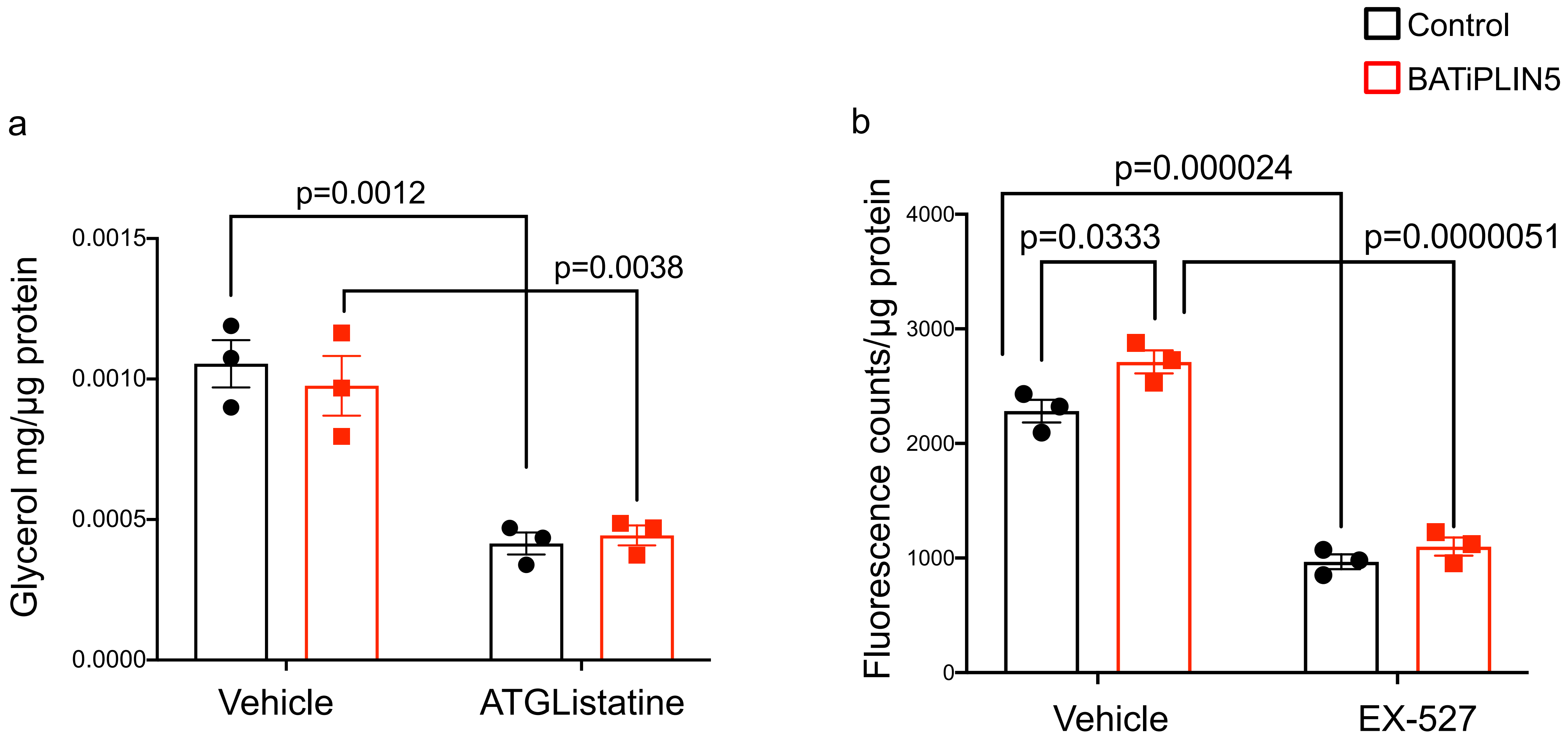

Supplementary Figure 16. Lipolysis assay and Sirt1 activity assay of BAT from BATiPLIN5 and Control mice

a. Ex vivo basal lipolysis (glycerol release) in minced BAT from Control or BATiPLIN5 mice treated with Vehicle or ATGListatine. *n*=3 per group.

b. Fluorescent Sirt1 activity assay in isolated mitochondria from Control or BATiPLIN5 mice treated with Vehicle or Ex-527. *n*=3 mice per group.

Values are mean ± s.e.m. Statistical analysis was performed using two-way ANOVA followed by Tukey post-test. P values are shown in the Figures. Source data are provided as a Source Data file.

Supplementary Figure 17

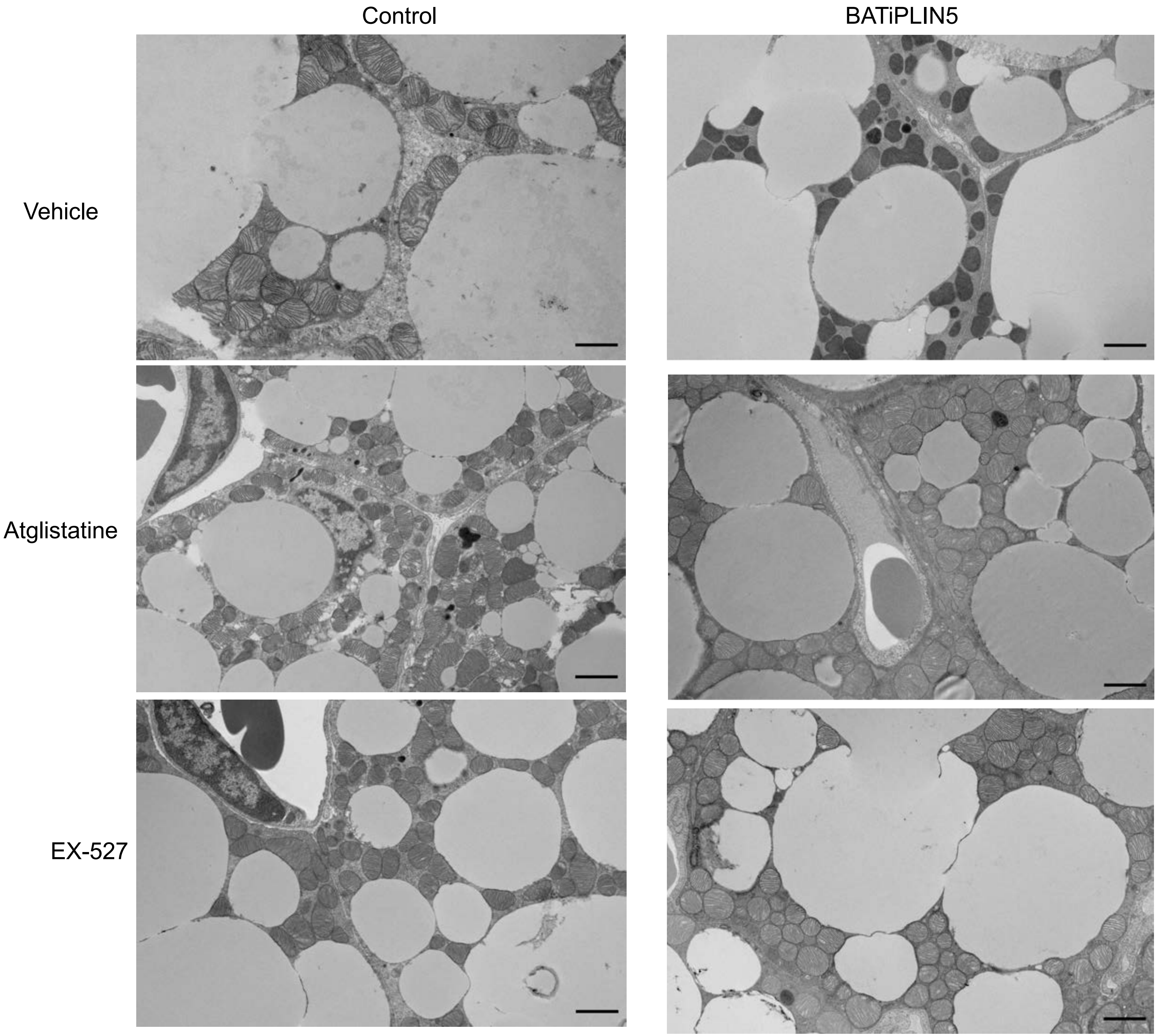

Supplementary Figure 17. Electron microscopy images of BAT from BATiPLIN5 and Control mice treated with Atglistatine or EX-527

a. Additional representative images of BAT electron microscopy (EM) from Control or BATiPLIN5 mice housed at 23 ºC and treated with Vehicle or ATGListatine or Ex-527. Scale bar= 2 μm.

Supplementary Figure 18

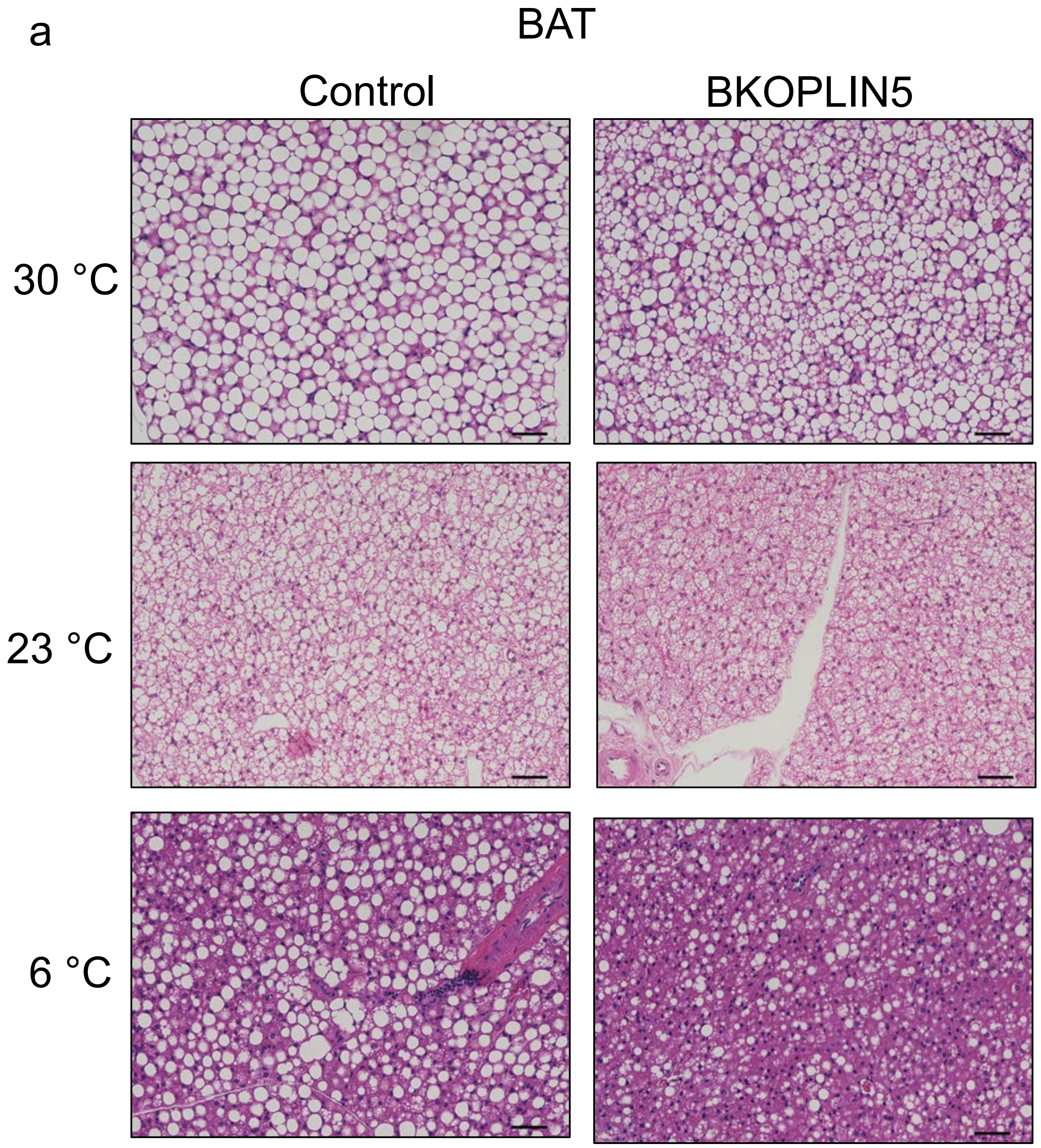

Supplementary Figure 18. Hematoxylin and eosin staining of BAT from BKOPLIN5 and Control mice

a. Additional representative images of hematoxylin and eosin staining of BAT from Control or BKOPLIN5 mice housed at 23 ºC or exposed overnight to 30 ºC or 6 ºC. Scale bar=100 μm.

Supplementary Figure 19

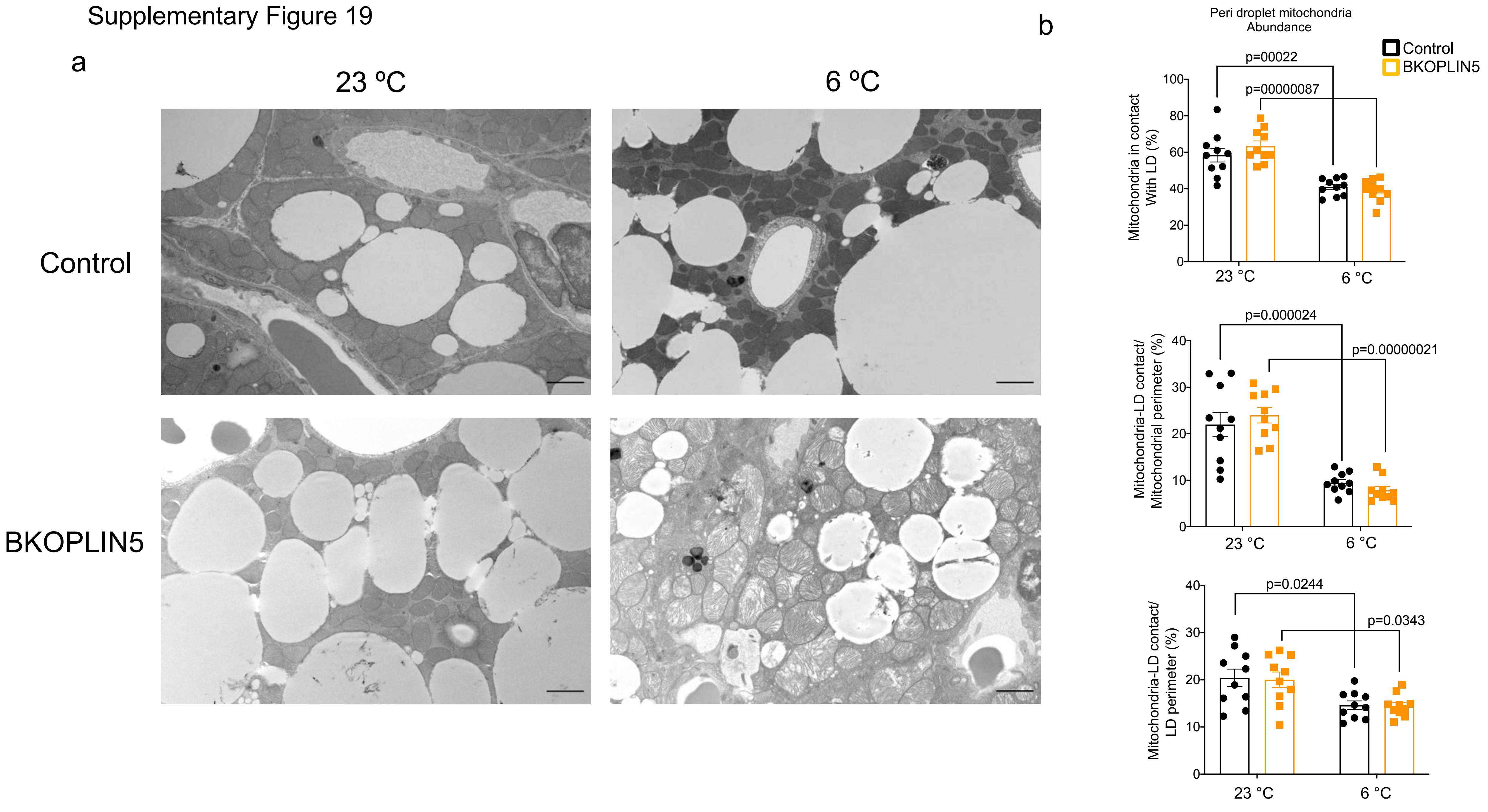

Supplementary Figure 19. Electron microscopy of and LD-mitochondria contact sites quantification of BAT from BKOPLIN5 and Control mice

a. Additional representative images of BAT electron microscopy from control or BKOPLIN5 mice housed at 23 ºC or exposed at 6 ºC for 16 hours. Scale bar= 2 µm.

b. Mitochondria in contact with lipid droplets quantified by count (top panel), % of mitochondrial perimeter (middle panel) and % of lipid droplet perimeter (bottom panel) *n*=10 EM fields per condition. Values are mean ± s.e.m. Statistical analysis was performed using two-way ANOVA followed by Tukey post-test. P values are shown in the Figures. Source data are provided as a Source Data file.

Supplementary Figure 20

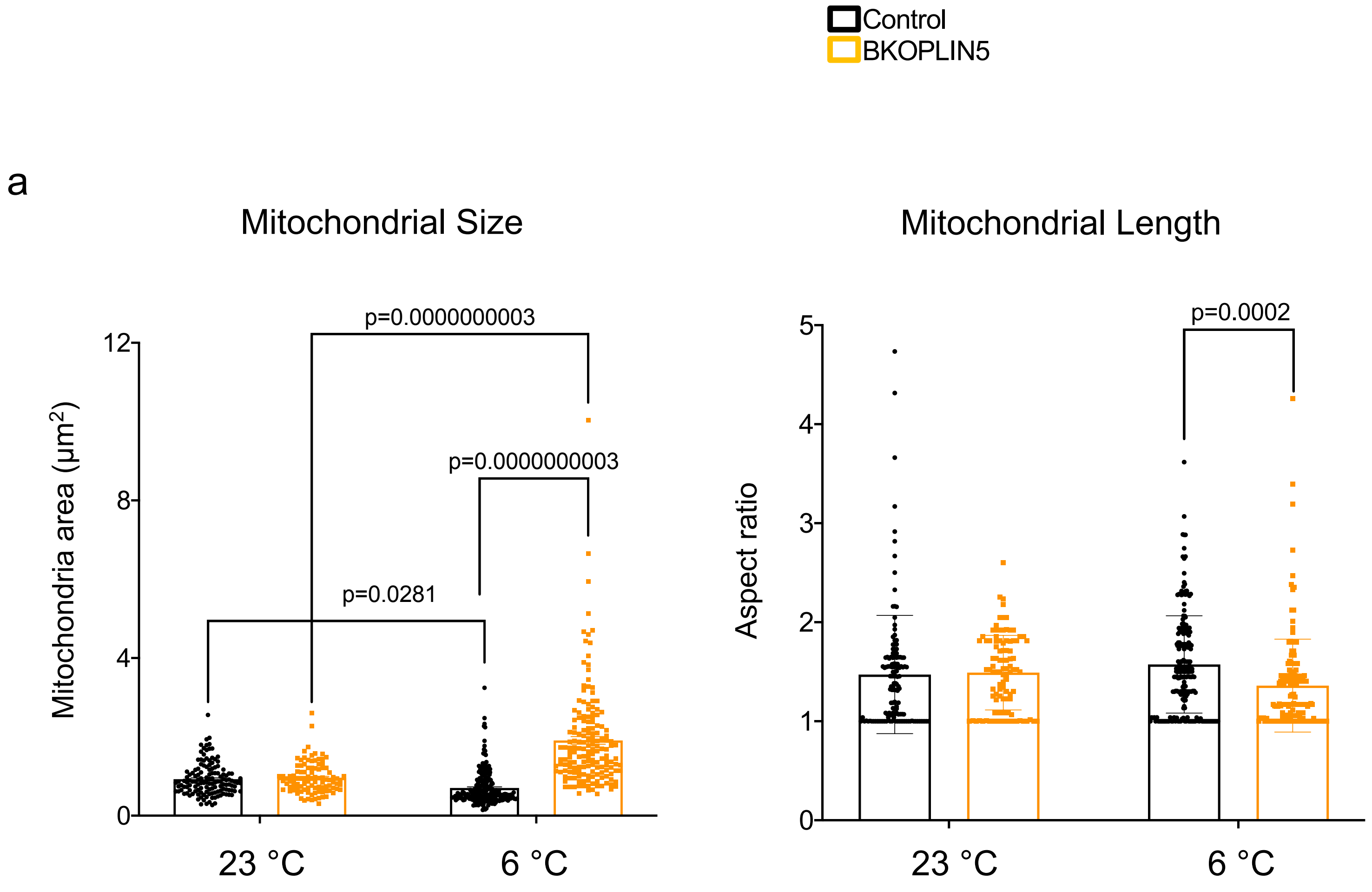

Supplementary Figure 20. Electron microscopy mitochondrial size quantification of BAT from BKOPLIN5 and Control mice

a. Mitochondrial area (left panel), mitochondrial length (right panel). *n*=137-220 mitochondria. Values are mean ± s.e.m. Statistical analysis was performed using two-way ANOVA followed by Tukey post-test. P values are shown in the Figures. Source data are provided as a Source Data file.

Supplementary Figure 21

a

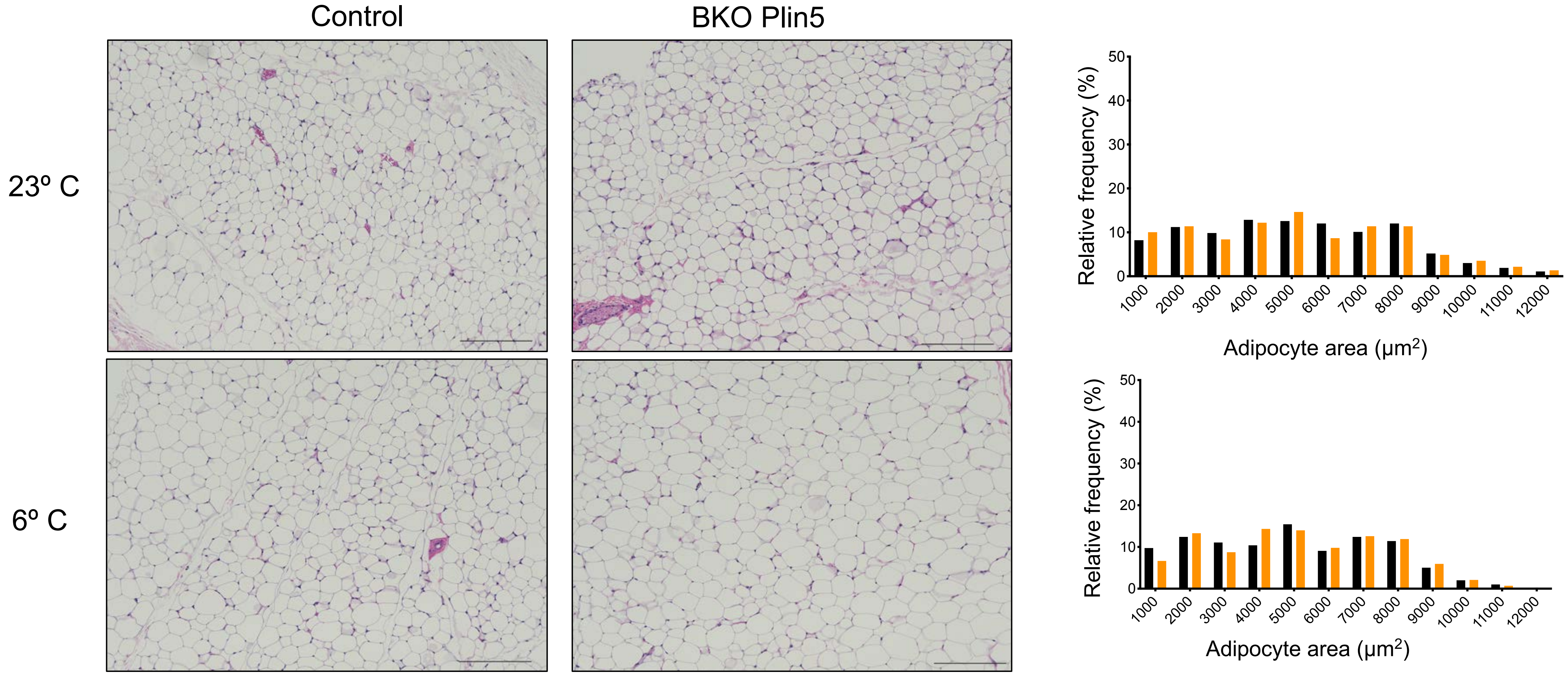

Supplementary Figure 21. Hematoxylin and eosin staining of iWAT from BKOPLIN5 and Control mice

a. Representative images of hematoxylin and eosin staining of white adipose tissue from Control or BKOPLIN5 mice housed at 23 ºC or exposed overnight to 6 ºC and adipocyte size quantification expressed as relative frequency . Scale bar=200 μm. Source data are provided as a Source Data file.
