# Supplementary material for: Perilipin 5 links mitochondrial uncoupled respiration in brown fat to healthy white fat remodeling and systemic glucose tolerance
Source: Nat Commun. 2021 Jun 3;12:3320. doi: 10.1038/s41467-021-23601-2 (PMC8175597; doi:10.1038/s41467-021-23601-2)
Supplement: Supplementary file 3 — Reporting Summary [file 41467_2021_23601_MOESM3_ESM.pdf]

nature research

Corresponding author(s): Perry E. Bickel

Last updated by author(s): March 1, 2021

Reporting Summary

Nature Research wishes to improve the reproducibility of the work that we publish. This form provides structure for consistency and transparency in reporting. For further information on Nature Research policies, see Authors & Referees and the Editorial Policy Checklist .

Statistics

For all statistical analyses, confirm that the following items are present in the figure legend, table legend, main text, or Methods section.

n/a	Confirmed	
☐	☒	The exact sample size (*n*) for each experimental group/condition, given as a discrete number and unit of measurement
☐	☒	A statement on whether measurements were taken from distinct samples or whether the same sample was measured repeatedly
☐	☒	The statistical test(s) used AND whether they are one- or two-sided<br>*Only common tests should be described solely by name; describe more complex techniques in the Methods section.*
☐	☒	A description of all covariates tested
☐	☒	A description of any assumptions or corrections, such as tests of normality and adjustment for multiple comparisons
☐	☒	A full description of the statistical parameters including central tendency (e.g. means) or other basic estimates (e.g. regression coefficient) AND variation (e.g. standard deviation) or associated estimates of uncertainty (e.g. confidence intervals)
☐	☒	For null hypothesis testing, the test statistic (e.g. *F*, *t*, *r*) with confidence intervals, effect sizes, degrees of freedom and *P* value noted<br>*Give P values as exact values whenever suitable.*
☒	☐	For Bayesian analysis, information on the choice of priors and Markov chain Monte Carlo settings
☒	☐	For hierarchical and complex designs, identification of the appropriate level for tests and full reporting of outcomes
☒	☐	Estimates of effect sizes (e.g. Cohen's *d*, Pearson's *r*), indicating how they were calculated

Our web collection on statistics for biologists contains articles on many of the points above.

Software and code

Policy information about availability of computer code

Data collection	For western blot band intensity analysis we used Image Studio Ver. 3.1 (Licor Biosciences). For qpcr Ct values analysis we used Quant Studio Real time qpcr software Ver. 3.1 (Applied Biosystems). Mitochondrial respiration data were analyzed using the Wave Desktop Software Ver. 2.6.1(Agilent technologies). For adipocyte area calculation we used Keyence BZ-X Analyzer software Ver. 1.3.03. For colorimetric microplate assays (protein quantification, glycerol, citrate synthase activity) we used Gen5 Ver 2.01.14 software. For micrograph analysis we used Image J NIH Ver. 1.53. For primer design we used Primer express 3.0.1 (Applied Biosystems) or Primer Blast (National Center for Biotechnology Information) Ver N/A
Data analysis	We used GraphPad Prism Ver. 8.0 to perform statistical analysis

For manuscripts utilizing custom algorithms or software that are central to the research but not yet described in published literature, software must be made available to editors/reviewers. We strongly encourage code deposition in a community repository (e.g. GitHub). See the Nature Research guidelines for submitting code & software for further information.

Data

Policy information about availability of data

All manuscripts must include a data availability statement. This statement should provide the following information, where applicable:

- Accession codes, unique identifiers, or web links for publicly available datasets
- A list of figures that have associated raw data
- A description of any restrictions on data availability

The data that supports the findings in this manuscript are included in the main figures and supplementary figures. All raw data are available from the corresponding author upon request

Field-specific reporting

Please select the one below that is the best fit for your research. If you are not sure, read the appropriate sections before making your selection.

[x] Life sciences [ ] Behavioural & social sciences [ ] Ecological, evolutionary & environmental sciences

For a reference copy of the document with all sections, see nature.com/documents/nr-reporting-summary-flat.pdf

Life sciences study design

All studies must disclose on these points even when the disclosure is negative.

Sample size

We determined sample size based on: 1) Previously published studies that used same or similar experimental methods (An, Y. A. et al. Nat Metab 1, 1243-1257, (2019). Heine, M. et al. Cell Metab 28, 644-655 e644, (2018). Li, X. et al. Molecular and cellular biology 40, doi:10.1128/MCB.00564-19 (2020). and 2)Pilot studies performed in the lab. Sample size is stated in each Figure legend

Data exclusions

No data exclusions

Replication

For reproducibility we performed independent experiments at least 2 times (two different cohorts of mice). Some experiments were independently reproduced more than 2 times. All the attemps of replication were succesful.

Randomization

Mice in the same cohort with different genotypes were randomly allocated to the indicated treatment (e.g. Chow vs HFD)

Blinding

During the execution of some experiments and when possible, investigators were blinded to genotype of the mice. For histology and electron microscopy, the investigator was blinded to mouse genotype during image acquisition. For experiments with chow or high fat diet, blinding was not possible due to obvious differences between diets. In some experiments, blinding was not possible, because the setup and execution of the experiment were performed by the same person.

Reporting for specific materials, systems and methods

We require information from authors about some types of materials, experimental systems and methods used in many studies. Here, indicate whether each material, system or method listed is relevant to your study. If you are not sure if a list item applies to your research, read the appropriate section before selecting a response.

Materials & experimental systems

n/a	Involved in the study	
[ ]	[x]	Antibodies
[x]	[ ]	Eukaryotic cell lines
[x]	[ ]	Palaeontology
[ ]	[x]	Animals and other organisms
[x]	[ ]	Human research participants
[x]	[ ]	Clinical data

Methods

n/a	Involved in the study	
[x]	[ ]	ChIP-seq
[x]	[ ]	Flow cytometry
[x]	[ ]	MRI-based neuroimaging

Antibodies

Antibodies used

Detailed information regarding how each antibody was used is included in the section Methods for Antibodies. anti guinea pig Perilipin 5 (Cat # GP31) was purchased from Progen. anti rabbit Perilipin 5 was generated by Bickel lab and detailed info can be found in Methods reference 1. anti-Ucp1 was purchased from Abcam (Cat # 209483). anti-Actin was purchased from Santa Cruz Biotechnology sc-47778. anti-GDI was generated by Perry Bickel and detailed information can be found in Methods reference 10. anti-GAPDH was purchased from Cell Signaling (Cat # 2118S), anti-total AKT was purchased from Santa Cruz Biotechnology (Cat # sc1619). anti-phosphoAKT was purchased from CellSignaling (Cat # 4060S). For secondary antibodies Donkey anti guinea pig 800CW Licor Cat # 926-32411, Goat anti Rabbit 680RD Licor Cat # 926-68071, Donkey anti Mouse 800CW Licor Cat # 925-32212, Donkey anti Goat 800 CW Licor Cat # 926-32214

Validation

According to vendor anti guinea pig Perilipin 5 (Progen) is validated to use in western blot and has been used in multiple publications (https://www.progen.com/ProductLeaflet/file/getpdf/name/anti-Perilipin_5_%28C-terminus%29_guinea_pig_polyclonal%2C_serum.pdf?fileId=454). Additionally we had tested this antibody using Perilipin 5 KO mouse and a specific band in the right size can be found in the control mice but not in the KO mice. anti rabbit Perilipin-5 (Bickel Lab) detailed information regarding validation and images can be found in Methods reference 1, additionally we have tested this antibody using Perilipin 5 KO mice and a specific band in the right size can be found in the control mice but not in the KO mice. According to vendor anti-Ucp1 (Abcam) has been validated to use in western blot (https://www.abcam.com/ucp1-antibody-epr20381-ab209483.html) and has been widely use in multiple publications. Additionally we have tested this antibody using the UCP1 KO mice and a specific band can be found in the control mice and not in the KO mice. anti-GDI was generated by Perry Bickel and detailed information can be found in Methods reference 10. According to vendor (Santa Cruz Biotechnology) anti-Actin has been validated to use in western blot and has been widely use in multiple publications (https://www.scbt.com/p/beta-actin-antibody-c4), anti-total AKT from Santa Cruz Biotechnology is currently unavailable but according to the vendor has been used in multiple publications (https://www.scbt.com/p/akt1-2-antibody-n-19?requestFrom=search). anti-phosphoAKT was purchased from

	CellSignaling and has been validated for western blot use. References can be found https://media.cellsignal.com/pdf/4060.pdf. Anti-GAPDH from Cell Signaling has been widely use and references can be found here https://www.cellsignal.com/products/primary-antibodies/gapdh-14c10-rabbit-mab/2118

Animals and other organisms

Policy information about studies involving animals; ARRIVE guidelines recommended for reporting animal research

Laboratory animals	For all experiments presented in this study, we used male mice on a C57BL/6J background. For all the experiments male mice were used. Unless otherwise indicated, we started the special diet 8 weeks after birth, and in general experiments were performed 12-16 weeks after birth (4-8 weeks after initiation of special diet). We housed mice in a conventional animal facility at 23 Celsius in a 12-h light/dark cycle with free access to food and water, unless otherwise indicated in the text, Figure Legends or Methods
Wild animals	No wild animals used in this study
Field-collected samples	No field collected samples in this study
Ethics oversight	We performed all animal experiments with approval from the University of Texas Southwestern Medical Center (UTSW) Institutional Animal Care and Use Committee (APN:2015-101325G).

Note that full information on the approval of the study protocol must also be provided in the manuscript.
